# No adverse association between exercise exposure and diffuse myocardial fibrosis in male endurance athletes

**DOI:** 10.1038/s41598-024-57233-5

**Published:** 2024-03-19

**Authors:** Kristoffer Andresen, Lars Gunnar Klæboe, Øyvind Haugen Lie, Kaspar Broch, Anette Borger Kvaslerud, Gerhard Bosse, Einar Hopp, Charlotte de Lange, Kristina Hermann Haugaa, Thor Edvardsen

**Affiliations:** 1 ProCardio Center for Innovation, Department of Cardiology, Oslo University Hospital, Rikshospitalet, Nydalen, P. O. Box 4950, N-0424 Oslo, Norway; 2 Faculty of Medicine, University of Oslo, Oslo, Norway; 3 Department of Cardiology, Akershus University Hospital, Lørenskog, Norway; 4 Division of Radiology and Nuclear Medicine, Oslo University Hospital, Rikshospitalet, Oslo, Norway; 5 Institution of Clinical Sciences, Sahlgrenska Academy, University of Gothenburg, Gothenburg, Sweden; 6 Department of Pediatric Radiology, Queen Silvia Children’s Hospital, Sahlgrenska University Hospital, Gothenburg, Sweden; 7 Heart and Lung Diseases Unit, Department of Medicine, Karolinska University Hospital, Huddinge, Sweden

**Keywords:** Cardiology, Cardiomyopathies

## Abstract

The potential association between endurance exercise and myocardial fibrosis is controversial. Data on exercise exposure and diffuse myocardial fibrosis in endurance athletes are scarce and conflicting. We aimed to investigate the association between exercise exposure and markers of diffuse myocardial fibrosis by cardiovascular magnetic resonance imaging (CMR) in endurance athletes. We examined 27 healthy adult male competitive endurance athletes aged 41 ± 9 years and 16 healthy controls in a cross sectional study using 3 Tesla CMR including late gadolinium enhancement and T1 mapping. Athletes reported detailed exercise history from 12 years of age. Left ventricular total mass, cellular mass and extracellular mass were higher in athletes than controls (86 vs. 58 g/m^2^, 67 vs. 44 g/m^2^ and 19 vs. 13 g/m^2^, all p < 0.01). Extracellular volume (ECV) was lower (21.5% vs. 23.8%, p = 0.03) and native T1 time was shorter (1214 ms vs. 1268 ms, p < 0.01) in the athletes. Increasing exercise dose was independently associated with shorter native T1 time (regression coefficient − 24.1, p < 0.05), but expressed no association with ECV. Our results indicate that diffuse myocardial fibrosis has a low prevalence in healthy male endurance athletes and do not indicate an adverse dose–response relationship between exercise and diffuse myocardial fibrosis in healthy athletes.

## Introduction

The beneficial effect of physical activity on reducing mortality and risk of cardiovascular disease in the general population is well established^1–3^. There is a non-linear dose–response relationship between physical activity and cardiovascular health. The most sedentary individuals benefit the most from increasing exercise doses^3^. However, the long-term cardiac consequences of elite athleticism and exercise doses far beyond what is recommended for the improvement and sustainment of cardiovascular health is still a matter of controversy.

It has long been hypothesized that excessive physical activity can induce permanent cardiac damage even in normal hearts^4^. Under this hypothesis, elite athleticism may in itself cause cardiac disease and not merely trigger arrhythmia in susceptible individuals. Consequent myocardial fibrosis can be either focal or diffuse, which can be visualised by cardiovascular magnetic resonance (CMR) imaging as areas of late gadolinium enhancement (LGE) and by T1 mapping as increased native T1 time and extracellular volume (ECV), respectively^5,6^. Both of these conditions have been found to be associated with increased mortality in clinical populations^7,8^. However, in athletes the prognostic significance of LGE is largely unknown^9–12^, whereas for T1 mapping it is uncharted.

Athletes have been found to have increased prevalence of LGE on CMR^12,13^, but not in general to be at increased risk of diffuse myocardial fibrosis^13–20^. However, studies utilizing CMR T1 mapping in athletes with LGE have been ambiguous, but may indicate a global myocardial involvement^14,15,19,21^. Further, there may be a dose–response relationship between exercise exposure and LGE in athletes^12,19,22–24^. However, no previous study has comprehensively investigated the association between exercise dose and diffuse myocardial fibrosis in athletes, and the limited available data on exercise history and its association with diffuse myocardial fibrosis in athletes are scarce and conflicting^18,25^.

In light of these observations, we hypothesize that there might be an association between exercise dose and diffuse myocardial fibrosis in athletes. The aim of this study was therefore to investigate the potential association between exercise and markers of diffuse myocardial fibrosis by CMR in healthy male competitive endurance athletes.

## Methods

### Study population

From April 2016 to February 2017, we enrolled 27 adult male competitive endurance athletes from Olympic to elite master level. Athletes were defined as subjects engaged in regular exercise training and participating in official sports competition^7^, and were required to have exercised for > six consecutive years at doses equivalent to > 24 metabolic equivalents of task (MET)-hours per week; three times the minimum recommended exercise dose for healthy adults^7,26^. The athletes were healthy and reported no use of prescription drugs or performance enhancing substances. We recruited 16 healthy male controls from adult blood donors aged 16 to 65 years. Disregarding the requirements of athletic status and history, controls were subject to the same inclusion and exclusion criteria as their athletic peers. We excluded individuals with signs or symptoms of cardiovascular disease, significant valvular or congenital heart disease, current comorbidity potentially influencing cardiovascular performance, antihypertensive or other vasoactive drug therapy or a body mass index (BMI) ≥ 30 kg/m^2^. The study complied with the Declaration of Helsinki and was approved by the Regional Committee for Medical and Health Research Ethics in South-Eastern Norway. All study participants gave written informed consent.

### Exercise history

All the athletes underwent a structured interview based on a questionnaire (Supplementary Fig. S1 and S2), retrospectively assessing their exercise history from 12 years of age. We defined exercise as any planned or structured action with the objective of improving or maintaining physical fitness or health^3^. Exercise duration was defined as the actual time in motion and accumulated exercise duration calculated as the sum of weekly durations from reported time intervals. Exercise intensity was estimated using the 2011 Compendium of Physical Activities and reported as MET defined as the ratio of the work metabolic rate to a standard resting metabolic rate of 1 kcal/kg/hour^26^. Exercise dose expressed as MET-hours was calculated as the product of exercise duration and exercise intensity^3^.

### CMR protocol

All subjects underwent CMR on the same 3 Tesla MRI scanner (Philips Ingenia; Philips Healthcare, Best, The Netherlands). ECG-gated balanced steady state free precession (bSSFP) two-dimensional cine sequences during breath hold were performed in standardized long axis and multiple short axis projections covering both ventricles from base to apex for anatomical and functional evaluation.

T1 mapping was performed in accordance with current recommendations^6^, using 5(3)3 modified Look-Locker Inversion recovery (MOLLI) sequences for native and 4(1)3(1)2 MOLLI sequences for post-contrast images.

A gadolinium-based contrast agent (0.2 mmol/kg of Dotarem/gadoterate meglumine; Guerbet, Aulnay-sous-Bois, France) was injected to perform post-contrast T1 mapping and evaluate the presence of LGE in the steady state ≥ 10 min after contrast administration.

### CMR analysis

Analyses of ventricular volumes and mass were performed semi-automatically and according to current recommendations^27^ in the bSSFP cine sequences using Segment version 3.2 R8531^28^. The endocardial and epicardial borders were delineated in end-diastole and end-systole for volumetric assessment paying careful attention to systolic basal descent of the atrioventricular plane. A constant of 1.05 g/ml for myocardial tissue was applied for calculation of left ventricular mass (LVM)^27^. Papillary muscles were excluded from the analysis of LVM to allow for comparison with reference values of left ventricular volumes and mass specific for endurance athletes^29^. Left ventricular cellular mass (CM) and extracellular mass (ECM) were calculated by Eqs. (1) and (2)^20^ as

1 $$CM=1-extracellular\, volume\, \left(ECV\right) \left(\%\right)\times left\, ventricular \,mass\, (LVM)$$

2 $$ECM=extracellular\, volume \,\left(ECV\right) \left(\%\right) \times left \,ventricular\, mass\, (LVM)$$

T1 mapping was performed in Sectra Picture Archive and Communication System (PACS) (Sectra AB, Linköping, Sweden), excluding any areas of LGE. ECV was calculated by Eq. (3)^30^ as

3 $$ECV (\%)= \frac{1/T1\,\left(myocardium\right)post \,contrast- 1/T1\left(myocardium\right)\,native}{1/T1\left(blood\right)\,post\, contrast- 1/T1\,\left(blood\right)\,native}\times\left(1-EVF\right)$$

EVF was derived from blood sampling in all but one athlete where synthetic ECV^31^ was reported with EVF derived from blood T1 due to missing data. Blood sampling was standardized and performed prior to the CMR examination after a minimum of 15 min of rest in a seated position, predominantly from the right antecubital vein.

Visual assessment of LGE was performed in Sectra PACS and semi-automatically quantified using Segment. The distribution of LGE was classified as insertion point, non-ischemic and ischemic.

### Statistics

Descriptive statistics are reported as mean ± standard deviation for parametric data, median (interquartile range, IQR) for non-parametric data and numbers (%) for categorical data. Between-group differences for continuous variables were evaluated using independent samples T-test for parametric data and independent samples Mann–Whitney U Test for non-parametric data. Pearson Chi-Square test was applied to compare distributions of categorical outcomes, whereas Fisher’s Exact test was applied in the case of low expected frequencies (< 5) in the analyses. The upper limit of normality (ULN) of the T1 mapping results were defined as the mean plus two standard deviations of the normal data^6^. Bivariate associations between exercise history data and CMR results were analysed calculating Spearman’s correlation coefficients due to non-parametric distribution of data. We used linear regression to assess associations between exercise history and CMR data, and adjusted for age, EVF and the presence of LGE in multivariable models. Accumulated exercise dose was chosen from the exercise metrics for the regression model as it expressed the strongest correlations to myocardial structure in univariable analysis and was the most comprehensive of the exercise metrics encompassing both duration and intensity over time. Assumptions of the linear regression models were investigated by histograms and normality plots of standardized residuals and plots of residuals versus fitted values. Associations between exercise history and the presence of LGE were assessed by logistic regression. Non-parametric distribution of the data were handled by logarithmic transformation or non-parametric tests. A two-tailed p-value of < 0.05 was considered statistically significant. Intra and inter observer variability was assessed by analysing intraclass correlation coefficients for native T1 time and ECV of ten study participants. Statistical analyses were performed using STATA version 17.

## Results

### Baseline characteristics and exercise history

Twenty-seven male athletes and 16 male controls were included in the study. The athletes had performed a median of 367 h of exercise per year since 12 years of age (Table 1). Five of the athletes were unwilling to detail their exercise history further than reporting average exercise intensity. The athletes had lower resting heart rate than the controls. There were no differences in body size or blood pressure between the groups. Importantly, there was no between-group difference in EVF, which is used for ECV calculation in the T1 mapping. The athletes had higher values of Troponin T than controls, where three of the athletes compared to none of the controls had values exceeding the upper reference limit of ≤ 14 ng/L for Troponin T (15, 22 and 80 ng/L).

**Table 1:** Baseline characteristics and exercise history.

	Athletes (*n* = 27)	Controls (*n* = 16)	P-value
Baseline characteristics			
Age, years	41 ± 9	41 ± 12	0.87
Height, cm	183 ± 5	181 ± 8	0.45
Weight, kg	78 ± 8	80 ± 12	0.62
BSA, m^2^	2.0 ± 0.1	2.0 ± 0.2	0.96
BMI, kg/m^2^	23.4 ± 1.7	24.2 ± 2.5	0.22
Systolic BP, mm Hg	122 ± 12	119 ± 13	0.40
Diastolic BP, mm Hg	71 ± 11	69 ± 9	0.71
Heart rate, bpm	50 ± 9	65 ± 11	< 0.01
Hemoglobin, g/dL	15.0 ± 0.8	14.9 ± 0.8	0.73
EVF	0.45 ± 0.03	0.43 ± 0.02	0.08
NT-proBNP, pg/mL	< 50 (< 50- < 50)	< 50 (< 50- < 50)	0.51
Troponin T, ng/L	7 (< 5–12)	< 5 (< 5- < 5)	< 0.01
Exercise history			
Exercise duration, h/y	367 (256–445)	–	–
Exercise intensity, MET	8.5 (8.5–10.5)	–	–
Exercise dose, MET-h/y	3123 (2228–4474)	–	–
Accumulated duration, h	10,122 (7306–15,750)	–	–
Accumulated dose, MET-h	92,001 (62,101–148,512)	–	–
Types of primary sports			
Bicycling, n (%)	17 (63)	–	–
Cross-country skiing, n (%)	8 (30)	–	–
Rowing, n (%)	1 (4)	–	–
Triathlon, n (%)	1 (4)	–	–

*BMI* body mass index, *BP* blood pressure, *BSA* body surface area, *EVF* erythrocyte volume fraction, *MET* metabolic equivalent of task, *NT-proBNP* N-terminal pro-B type natriuretic peptide.

P-values for independent samples T-test for parametric data and Mann–Whitney U-test for non-parametric data.

### Cardiovascular magnetic resonance imaging

All athletes and controls underwent CMR. One athlete was excluded from the LGE and ECV calculations due to lack of contrast administration and two athletes were excluded from the T1 mapping analysis due to inadequate image quality in the T1 sequences and biologically implausible native T1 time (889 ms).

### Cardiac dimensions and function

The left and right ventricles were larger in the athletes than the controls (Table 2). Seven (26%) of the athletes and none of the controls exceeded the ULN for endurance athletes of indexed left and right ventricular end-diastolic volumes (ULN 121 ml/m^2^ and 126 ml/m^2^ respectively, p = 0.04 for difference). Total left ventricular mass was higher in athletes than controls. Twenty-five (93%) of the athletes and one (6%) of the controls had LVM exceeding the ULN for endurance athletes (ULN 76 g/m^2^, p < 0.01 for difference). Both cellular and extracellular mass were higher in the athletes than in the controls, and the ratio of cellular to extracellular mass was also higher in the athletes.

**Table 2:** Cardiac magnetic resonance imaging results.

	Athletes (*n* = 27)	Controls (*n* = 16)	P-value
Volumes and function			
LVEDV, ml/m^2^	114 ± 17	80 ± 10	< 0.01
LVESV, ml/m^2^	49 ± 11	35 ± 6	< 0.01
LVSV, ml/m^2^	64 ± 11	45 ± 6	< 0.01
LVEF, %	57 ± 6	56 ± 4	0.80
RVEDV, ml/m^2^	120 ± 19	95 ± 15	< 0.01
RVESV, ml/m^2^	59 ± 13	43 ± 9	< 0.01
RVSV, ml/m^2^	62 ± 10	52 ± 6	< 0.01
RVEF, %	52 ± 6	55 ± 4	0.02
CI, l/min/m^2^	3.2 ± 0.6	3.2 ± 0.4	0.96
LV mass			
LVM, g/m^2^	86 (81–98)	58 (53–61)	< 0.01
Cellular, g/m^2^	67 (62–74)	44 (40–46)	< 0.01
Extracellular, g/m^2^	19 (17–21)	13 (13–14)	< 0.01
CM:ECM ratio	3.7 (3.2–3.8)	3.2 (3.0–3.5)	0.03
LGE			
LGE, n (%)	5 (19)	0 (0)	0.14
Insertion point, n (%)	2 (8)	0 (0)	–^a^
Non-ischemic, n (%)	3 (12)	0 (0)	–^a^
Ischemic, n (%)	0 (0)	0 (0)	–^a^
T1 mapping			
Native T1 IVS, ms	1214 ± 24	1268 ± 48	< 0.01
ECV IVS, %	21.5 (20.8–23.8)	23.8 (22.0–25.2)	0.03

*CI* cardiac index, *CM* cellular mass, *ECM* extracellular mass, *ECV* extracellular volume, *EDV* end diastolic volume, *EF* ejection fraction, *ESV* end systolic volume, *IVS* interventricular septum, *LGE* late gadolinium enhancement, *LV* left ventricle, *LVM* left ventricular mass, *RV* right ventricle, *SV* stroke volume.

P-values for independent samples T-test for parametric data, Mann–Whitney U-test for non-parametric data and Fischer’s exact test for categorical data.

^a^Statistical significance testing not performed in subgroups of LGE due to low frequency.

### Late gadolinium enhancement

LGE was observed in five (19%) of the athletes and in none of the controls. The LGE was localized in the right ventricular posterior septal insertion point in two (8%) athletes and as non-ischemic LGE in three (12%) athletes. None of the athletes or controls had LGE suggestive of ischemia. The median extent of LGE in those five athletes was 1.0 (0.7–4.0) % of left ventricular mass. There were no clear differences between athletes with or without LGE in regards to exercise history, and no association between exercise history and presence of LGE (Table 3).

**Table 3:** Exercise history and presence of LGE.

	LGE + athletes	LGE- athletes	P-value for difference^a^	Logistic regression		
	*(n* = *5)*	*(n* = *21)*		OR^b^	95% CI	P-value
Exercise duration, h/y	309 (217–541)	367 (262–445)	0.77	1.00	(0.50, 1.98)	1.00
Exercise intensity, MET	8.5 (8.5–10.0)	8.5 (8.5–10.5)	1.00	1.14	(0.39, 3.31)	0.81
Exercise dose, MET-h/y	2625 (1846–5132)	3151 (2307–4475)	0.65	1.01	(0.53, 1.96)	0.97
Accumulated duration, h	9783 (5005–16,598)	10,122 (7852–15,750)	0.77	0.98	(0.81, 1.18)	0.80
Accumulated dose, MET-h	83,151 (42,543–156,079)	92,001 (66,742–148,512)	0.77	1.00	(0.98, 1.02)	0.84

*LGE* late gadolinium enhancement, *MET* metabolic equivalent of task.

^a^P-value for independent samples Mann–Whitney U Test.

^b^OR for LGE per 100 h change in exercise duration/year, 1 MET change in exercise intensity, 1000 MET-hours change in exercise dose/year, 1000 h change in accumulated exercise duration and 10.000 MET-hours change in accumulated exercise dose.

### T1 mapping

**Figure 1:**
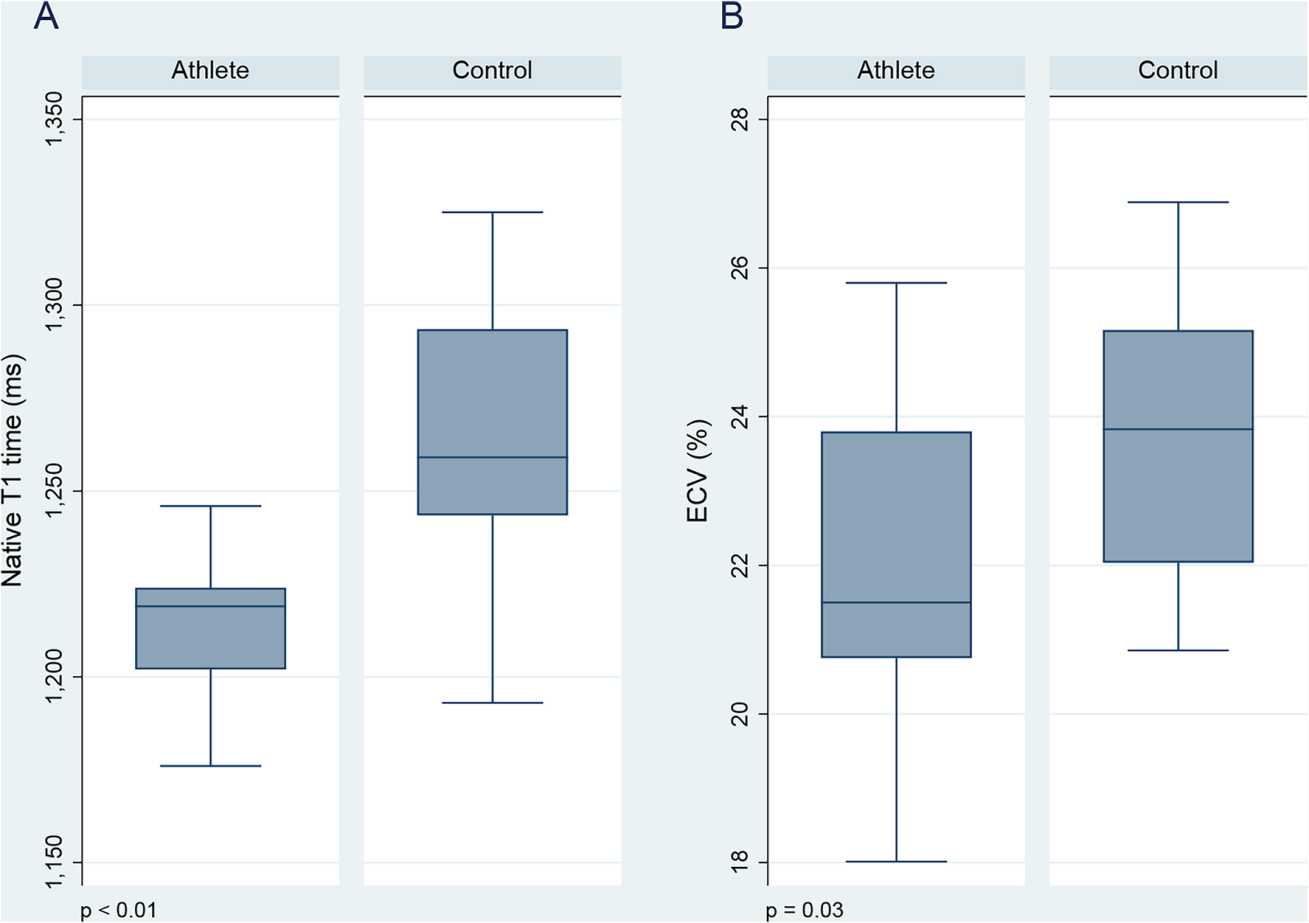
Native T1 time and ECV in athletes and controls. Boxplots of (**A**) native T1 time and (**B**) ECV demonstrating shorter native T1 time and lower ECV in the athletes. *ECV* extracellular volume.

**Figure 2:**
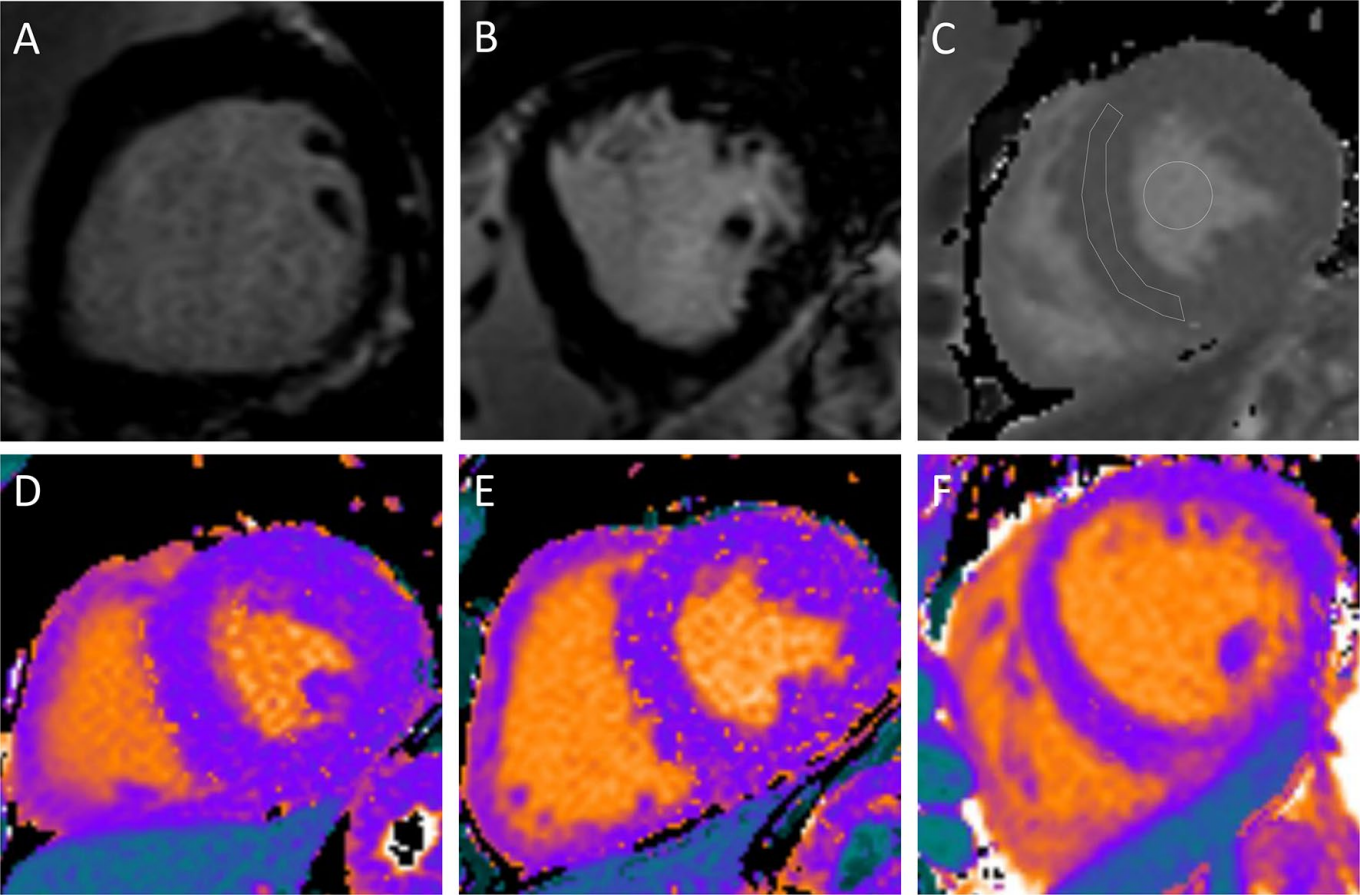
LGE and native T1 mapping by CMR. Illustrations of athletes with (**A**) non-ischemic LGE and (**B**) LGE in the right ventricular inferior insertion point. (**C**) Native T1 mapping with regions of interest. Colour-coded native T1 maps in (**D**) LGE-negative athlete: native T1 time 1204 ms, ECV 20.4%, (**E**) LGE-positive athlete: native T1-time 1222 ms, ECV 21.0% and (**F**) healthy control: native T1 time 1259 ms, ECV 24.3%. *ECV* extracellular volume, *LGE* late gadolinium enhancement.

Athletes had shorter native T1 time (1214 ms vs. 1268 ms, p < 0.01) and lower ECV (21.5% vs. 23.8%, p = 0.03) in the interventricular septum than controls (Fig. 1). There were no differences in native T1 time (1220 ms vs. 1212 ms, p = 0.56) or ECV (21.5% vs. 21.5%, p = 0.89) between athletes with or without LGE. Native T1 mapping in athletes with and without LGE and a healthy control is illustrated in Fig. 2.

None of the athletes had abnormally long native T1 times, whereas two of the athletes had ECV exceeding the ULN (29.6% and 31.4% vs. 27.8%). Besides older age (49 and 47 years vs. mean 41 years), there were no clear differences between these two and the other athletes regarding baseline characteristics, exercise history or cardiac dimensions and function. None of the two athletes displayed LGE.

### Exercise history, T1 mapping and left ventricular mass

**Figure 3:**
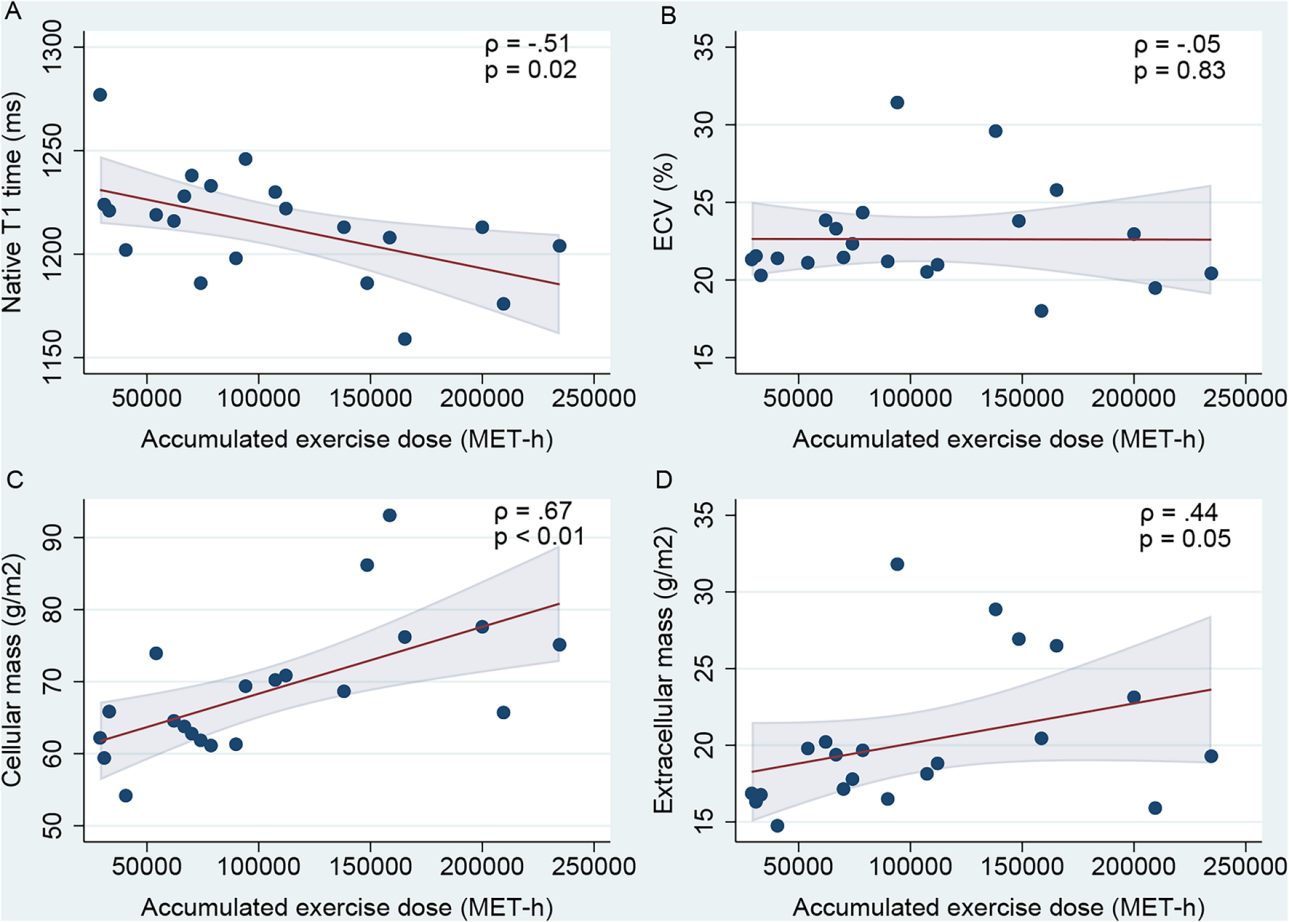
Exercise dose and myocardial structure by CMR. Scatterplots of accumulated exercise dose and (**A**) native T1 time, (**B**) ECV, (**C**) left ventricular cellular mass and (**D**) left ventricular extracellular mass. *ρ* Spearman’s rho, *ECV* extracellular volume, *MET* metabolic equivalent of task.

Accumulated, but not average, exercise duration and dose were negatively associated with the native T1 time (Table 4). None of the parameters of exercise exposure were associated with ECV. All parameters of exercise exposure were associated with left ventricular total mass and left ventricular cellular mass, but there was no association between exercise exposure and left ventricular extracellular mass. The associations between accumulated exercise dose and native T1 time, ECV and left ventricular cellular and extracellular mass are demonstrated in Fig. 3.

**Table 4:** Bivariate correlations between exercise exposure and myocardial structure.

	Native T1, ms	ECV, %	LVM, g/m^2^	CM, g/m^2^	ECM, g/m^2^
Exercise duration, h/y	− 0.30	0.00	0.50^a^	0.50^a^	0.36
Exercise dose, MET-h/y	− 0.41	0.06	0.54^b^	0.55^b^	0.40
Accumulated duration, h	− 0.49^a^	− 0.07	0.66^c^	0.65^c^	0.43
Accumulated dose, MET-h	− 0.51^a^	− 0.05	0.66^c^	0.67^c^	0.44

*CM* cellular mass, *ECM* extracellular mass, *ECV* extracellular volume, *LVM* left ventricular mass, *MET* metabolic equivalent of task.

All correlation coefficients are reported as Spearman’s ρ due to non-parametric distribution of data.

^a^p < 0.05.

^b^p = 0.01.

^c^p = 0.001.

The accumulated exercise dose was negatively associated with the native T1 time and positively associated with all three parameters of left ventricular mass, but not ECV (Table 5). In multivariable analysis adjusting for age, EVF and presence of LGE, accumulated exercise dose remained negatively associated with the native T1 time and positively associated with total left ventricular mass and left ventricular cellular mass. There was no independent association between accumulated exercise dose and left ventricular extracellular mass.

**Table 5:** Linear regression models of accumulated exercise dose on myocardial structure.

	Univariable regression			Multivariable regression^a^		
	Regr. Coeff	95% CI	P-value	Regr. Coeff	95% CI	P-value
Native T1, ms	− 21.9	(− 38.1, − 5.6)	0.01	− 24.1	(− 47.3, − 0.9)	0.04
lnECV, %	0.0	(− 0.1, 0.1)	0.72			
lnLVM, g/m^2^	0.14	(0.06, 0.21)	< 0.01	0.13	(0.03, 0.23)	0.01
lnCM, g/m^2^	0.13	(0.06, 0.20)	< 0.01	0.13	(0.04, 0.23)	0.01
lnECM, g/m^2^	0.16	(0.02, 0.29)	0.03	0.15	(− 0.06, 0.35)	0.14

*ECM* extracellular mass, *ECV* Extracellular volume, *LVM* left ventricular mass, *CM* cellular mass.

^a^Adjusted for age, EVF and presence of LGE.

### Reproducibility

Intraclass correlation coefficients (ICC) for native T1 time and ECV were excellent for both intra and inter observer reanalysis at 0.95 and 0.99 for native T1 time and 0.99 and 0.98 for ECV, respectively (all p < 0.01).

## Discussion

Our study is the first to examine the association between exercise dose and diffuse myocardial fibrosis in endurance athletes using CMR. We did not find an association between athleticism and diffuse myocardial fibrosis. Our main results were: (1) athletes had shorter native T1 time and lower ECV than controls; (2) there were moderate inverse associations between exercise exposure and native T1 time, and there was no association between exercise exposure and ECV. Our results seem therefore to oppose the hypothesis of exercise-induced diffuse myocardial fibrosis.

### Native T1 time and ECV

Our observation of shorter native T1 time and lower ECV in athletes than controls is in contrast to what one would expect in exercise-induced diffuse myocardial fibrosis. These results indicate that diffuse myocardial fibrosis is not prevalent in healthy male endurance athletes despite a high cumulative exercise load and a marked expression of the athlete’s heart phenotype. Our results are in line with most previous CMR studies on endurance athletes, showing either neutral results or shorter native T1 time and lower ECV in athletes^14–17,19,20^. ECV is a validated surrogate marker of diffuse myocardial fibrosis in the absence of infiltration or acute myocardial injury, and ECV is strongly related with histology^6,32^. Higher ECV is associated with adverse outcomes in clinical populations^8^, but the prognostic value of T1 mapping in athletes is unknown.

### Left ventricular mass

The athletes had higher left ventricular mass than controls. As expected, cellular as well as extracellular mass was enlarged. However, the cellular enlargement was more pronounced than the extracellular enlargement which is reflected in the lower ECV observed in the athletes in our study. This suggests that the physiologic remodelling of the left ventricular myocardium observed in the athlete’s heart is predominantly caused by myocyte hypertrophy with a relatively smaller increase of the interstitium. Our results are in apparent contrast to what one would expect in diffuse myocardial fibrosis and in line with previous CMR studies on athletic remodelling observing greater relative expansion of the cellular than the extracellular compartment of the myocardium in athletes^16,20^.

Our results also show that athletes with the largest exercise exposure have the greatest degree of athletic remodelling. This is in line with the current perception of the athlete’s heart^33^. However, only the left ventricular cellular mass was independently associated with exercise exposure, whereas the extracellular mass was not. These results might indicate that there is a dose–response relationship between exercise exposure and left ventricular cellular expansion in healthy male endurance athletes, but probably not for extracellular mass.

In summary, our observations regarding exercise exposure and myocardial remodelling indicate that the enlargement of left ventricular mass in the athlete’s heart phenotype is accompanied by a relative expansion of the cellular compartment of the myocardium with a dose–response relationship between exercise exposure and myocyte hypertrophy.

### Exercise exposure and diffuse myocardial fibrosis

Exercise exposure was independently associated with shorter native T1 time and expressed no association with ECV in our athletes. This contradicts the supposition that exercise may cause diffuse myocardial fibrosis, where native T1 time and ECV would be expected to increase^6^. Existing data on the association between exercise exposure and diffuse myocardial fibrosis in athletes are scarce and limited to one study on veteran runners which found no association between total training volume and ECV^18^ and another study on a mixed group of athletes which found a possible association between increasing number of exercise years and longer native T1 time^25^. Whereas the former is in line with results from our study, our results contradict the latter. However, no previous study has comprehensively evaluated the association between exercise dose and diffuse myocardial fibrosis in endurance athletes using CMR. Our results indicate that exercise is not adversely associated with diffuse myocardial fibrosis.

### Late gadolinium enhancement

We observed LGE in five of the athletes in our study and in none of the controls. Several studies^14–19,21–24,34,35^ and reviews^9,12,13^ have addressed the issue of LGE in athletes during the recent years reporting prevalences ranging from 0 to 50% in often small-scale studies. Hence, the prevalence of LGE in the athletes in our study is comparable to previous literature. Multiple pathophysiological mechanisms for this have been proposed, including genetic predisposition, pulmonary artery pressure and/or volume overload, myocarditis, myocardial ischemia and repetitive microdamage from repeated bouts of intense exercise^12–15,17–19,21–23,34^. The LGE has typically been distributed in three different patterns: right ventricular septal insertion point, non-ischemic and ischemic^13^. Some studies have suggested a dose–response relationship between exercise exposure and LGE in athletes^12,19,22–24^, but the evidence is conflicting^14,18,21,35^. We found no difference in exercise history between athletes with or without LGE, nor any association between exercise history and presence of LGE. However, as the study sample was quite small and the number of athletes with LGE limited, these neutral results should be interpreted with caution as further evaluation of the potential association between exercise metrics and LGE was beyond the scope of this study to address.

Interestingly, there were no differences in native T1 time or ECV between athletes with or without LGE in our study sample, suggesting that LGE in these athletes was not associated with diffuse fibrosis. There have been conflicting results in the literature regarding this issue where some studies have reported higher ECV in the remote myocardium of athletes with LGE^19,21^, whereas other studies have found no such results^14,15^.

The presence of LGE is a risk factor for adverse outcomes in clinical populations, but the prognostic implication of non-specific LGE in asymptomatic athletes is uncertain^9–12^. However, it may not be a benign phenomenon as higher prevalence of LGE have been found in athletes evaluated for ventricular arrhythmia compared to asymptomatic athletic controls^36,37^ with reduced survival free from major arrhythmic events in LGE positive athletes^37^. In pathology studies, myocardial fibrosis has been attributed to up to 6% of cases of sudden cardiac death (SCD) in athletes^38^.

### Athlete’s heart

The athletes in our study displayed the characteristic phenotype of the athlete’s heart^39^ including symmetrical chamber enlargement, enlarged left ventricular mass and lower resting heart rate compared to controls. The extent of remodelling may be exemplified by 26% of athletes exceeding the upper reference values for ventricular volumes and 93% for left ventricular mass when comparing with proposed reference values for dimensional parameters in the male athlete’s heart^29^. As a profound degree of remodelling would be expected in these athletes considering their participation in competitive endurance sports at Olympic to elite master level, we perceive this extent of remodelling as an indicator of the sample being representative of the study population.

### Troponin T

Baseline characteristics in athletes and controls excluding athletic traits were well matched. We did, however, observe a difference in Troponin T values between athletes and controls where three of the athletes and none of the controls exceeded the upper reference limit for Troponin T for our laboratory. Athletes were not detrained prior to investigation, hence this could be explained by post exercise rise in cardiac troponins which is a well-known and usually physiologic phenomenon^40^. Although being asymptomatic, this could alternatively be a sign of sub-clinical cardiac disease in these athletes. The cross-sectional study design with no follow-up data did not permit further investigation of this observation.

### Strengths and limitations

This was a single-centre, observational study with inherent limitations in regards to external validity and causal inference. We recruited healthy athletes only, which may have introduced a selection bias. Our study sample is small, leaving the results prone to type-2 error. Also, we evaluated male athletes only. Our results therefore do not necessarily translate to women.

Exercise exposure was evaluated retrospectively as this was considered to be the most feasible option. All the athletes were actively competitive, well-performing and asymptomatic, and we do not consider the exercise history data of the athletes to be subject to bias beside the inherent potential of inaccuracy of retrospective data. We did not evaluate the exercise history of the control group as this would have introduced a potential for recall bias in the data due to expected differences in interest regarding exercise and sports between the groups. The lack of exercise history for the control group could have introduced a risk of type-2 error if the controls also had been exercising extensively. However, the groups were distinctively different based on the profound expression of the athlete’s heart phenotype in the athlete group; hence there is no evidence suggestive of this in our data.

We performed cardiac magnetic resonance imaging using a 3 Tesla MRI scanner. Whereas T1 and ECV mapping are typically performed either at 1.5 Tesla or 3 Tesla scanners^6^, parametric mapping results obtained by different magnetic field strengths cannot be used interchangeably^41^. Higher magnetic field strength in 3 Tesla CMR results in a higher signal-to-noise ratio, contrast-to-noise ratio and may improve the precision of T1 mapping, but has disadvantages due to increased energy deposits and potential loss of homogeneity in the magnetic and radiofrequency fields^41^. There is no clear preference of either field strength in CMR T1 mapping^41^, but the choice of field strength will directly affect the absolute values of the parametric mapping results as the T1 relaxation time increases with increasing strength of the magnetic field^41^. However, this is only one amongst several sources of variability in CMR parametric mapping as relaxation times also vary between vendors, scanners and pulse sequences^6^. Hence, the methodology of CMR T1 mapping is highly dependent on the application of local reference values in the interpretation of results and participants being examined under similar conditions^6,41^, which we have taken great care to optimize in this study.

Correct ECV calculation is dependent on standardization of blood sampling for EVF determination to reduce variability. We ensured that blood sampling of all study participants were performed under similar conditions to avoid bias. Potential random variability might have been further reduced by performing blood sampling in the supine position as postural changes in EVF may influence ECV calculation^42^.

Finally, we have only performed T1 mapping of the left ventricular myocardium, whereas it is the right ventricle which experiences the greatest acute impact of exercise and displays the greatest degree of chronic remodelling in response to exercise^22,43^. Parametric mapping of the right ventricular myocardium is inherently challenging due to the thin wall of the right ventricle and risk of partial volume effects^5^.

We would encourage future studies on athletic remodelling and myocardial fibrosis to report exercise dose to further increase the external validity of our results across different CMR conditions and study samples, as well taking potential gender differences into account. There is also a need for prospective data on the long-term consequences of exercise-induced cardiac remodelling which was beyond the scope of this study to address, but which might be remedied by the ongoing multicentre Pro@Heart cohort study^44^.

## Conclusions

Healthy male endurance athletes had lower ECV and shorter native T1 time than controls despite high cumulative exercise exposure and a profound expression of the athlete’s heart phenotype. Our results indicate that diffuse myocardial fibrosis is not prevalent in healthy male endurance athletes. Moreover, we did not find any adverse dose–response relationship between exercise exposure and diffuse myocardial fibrosis. These results offer reassurance and might help mitigate concerns regarding the potential for adverse long-term cardiac remodelling of elite athleticism in healthy athletes.

### Supplementary Information

Supplementary Figures.

## Data Availability

The data underlying this article cannot be shared publicly due to the privacy of individuals that participated in the study. The data will be shared on reasonable request to the corresponding author.
