# Supplementary material for: No adverse association between exercise exposure and diffuse myocardial fibrosis in male endurance athletes
Source: Sci Rep. 2024 Mar 19;14:6581. doi: 10.1038/s41598-024-57233-5 (PMC10951320; doi:10.1038/s41598-024-57233-5)
Supplement: Supplementary file 1 — Supplementary Figures. [file 41598_2024_57233_MOESM1_ESM.pdf]

Supplementary Information

Title

No adverse association between exercise exposure and diffuse myocardial fibrosis in male endurance athletes

Author information

Kristoffer Andresen[1, 2], Lars Gunnar Klæboe[1, 3], Øyvind Haugen Lie[1], Kaspar Broch[1, 2], Anette Borger Kvaslerud[1, 2], Gerhard Bosse[4], Einar Hopp[4], Charlotte de Lange[5, 6], Kristina Hermann Haugaa[1, 2, 7], Thor Edvardsen* [1, 2]

Affiliations

[1] ProCardio Center for Innovation, Department of Cardiology, Oslo University Hospital, Rikshospitalet, Oslo, Norway.

[2] Faculty of Medicine, University of Oslo, Oslo, Norway.

[3] Department of Cardiology, Akershus University Hospital, Lørenskog, Norway.

[4] Division of Radiology and Nuclear Medicine, Oslo University Hospital, Rikshospitalet, Oslo, Norway.

[5] Institution of Clinical Sciences, Sahlgrenska Academy, University of Gothenburg, Gothenburg, Sweden.

[6] Department of Pediatric Radiology, Queen Silvia Children's Hospital, Sahlgrenska University Hospital, Gothenburg, Sweden.

[7] Heart and Lung Diseases Unit, Department of Medicine, Karolinska University Hospital, Huddinge, Sweden.

* Corresponding author

OSLO UNIVERSITY HOSPITAL

NAME: **DATE OF BIRTH:**

Questionnaire on exercise from school age.

State physical activity from school age, where exercise was the purpose.
-If multiple activities – use multiple lines. E.g. football, swimming, running.
-Level/intensity: 1 – low intensity (sweaty forehead). 2- moderate intensity (exercise – shortness of breath). 3- high intensity (competition).
If level/intensity has changed over time, use a new line. E.g. 10-14 years swimming at moderate intensity, 15-19 years swimming at competitive intensity.
-Estimate average time spent per week for each activity.
-Estimate number of months per year you have performed each activity (e.g. cross-country skiing 5 months/year, football 8 months/year).
-If you have not been performing exercise, write «none» in the first line of the form.

Type of activity	Level/intensity	Age start	Age stop	Hours per week	Months per year	Remarks
Swimming	*2* EXAMPLE	*8 years*	*15 years*	*2*	*9*	*Not in summer vacation*
Brisk walks in the forest	*1*	*32 years*	*Ongoing*	*3*	*10*	*Walking uphill*

Supplementary Figure S1. Questionnaire on exercise from school age, English translation.

Oslo universitetssykehus

NAVN: **FØDSELSDATO:**

Spørreskjema om trening fra skolealder.

Angi fysisk aktivitet fra skolealder, hvor trening var formålet.
-Dersom flere forskjellige aktiviteter- bruk flere linjer. F.eks. fotball, svømming, løping.
-Nivå/intensitet: 1- lett aktivitet (svett på panne). 2- moderat intensitet (mosjon- kortpustet). 3- meget høy intensitet (konkurranse-intensitet).
Dersom nivå/intensitet er endret over tid, bruk ny linje. F.eks. fra 10-14 år svømming moderat intensitet, fra 15-19 år svømming konkurransenivå.
-Anslå gjennomsnittlig tidsbruk per uke for hver aktivitet.
-Anslå cirka hvor mange måneder i løpet av året du har vært aktiv med hver aktivitet (f.eks. ski 5 mnd per år, fotball 8 mnd/år).
-Dersom du ikke har drevet noen trening, skriv «ingen» i skjemaets første linje.

Type aktivitet	Nivå/intensitet	Alder start	Alder slutt	Cirka timer per uke	Måneder per år	Merknader
Svømming	*2* EKSEMPEL	*8 år*	*15 år*	*2*	*9*	*Fri sommerferie*
Raske gåturer i skogen	*1*	*32 år*	*Pågår*	*3*	*10*	*Går i motbakker*

Supplementary Figure S2. Questionnaire on exercise from school age, original version in Norwegian.
